# IS RDW A PREDICTIVE PARAMETER FOR CUBITAL TUNNEL SYNDROME PATIENTS REQUIRING SURGERY?

**DOI:** 10.1590/1413-785220162404156646

**Published:** 2016

**Authors:** Hakan Sarman, Cengiz Isik, Mehmet Boz, Ismail Boyraz, Bunyamin Koc, Sule Aydin Turkoglu

**Affiliations:** 1. Abant Izzet Baysal University, School of Medicine, Department of Orthopaedics and Traumatology, Bolu, Turkey; 2. Abant Izzet Baysal University, School of Medicine, Department of Physical Medicine and Rehabilitation, Bolu, Turkey; 3. Abant Izzet Baysal University, School of Medicine, Department of Neurology, Bolu, Turkey.

**Keywords:** Cubital tunnel syndrome/diagnosis, Cubital tunnel syndrome/etiology, Cubital tunnel syndrome/surgery, Cubital tunnel syndrome/blood

## Abstract

**Objective:**

The aim of this study was to investigate whether haemogram parameters are predictive factors for both the severity of the disease and a decision in favor of surgical treatment in patients with an established diagnosis of cubital tunnel syndrome (CuTS)***.***

**Methods:**

The medical files of patients with a diagnosis of CuTS who were followed-up conservatively (n=92) or surgically treated (n=92) were retrospectively screened and the haemogram parameters were recorded***.***

**Results:**

The receiver operating characteristic (ROC) curve analysis revealed an area of 0.665 under the curve, with 76.3% sensitivity and 84.8% specificity at the cut-off of a red cell distribution width (RDW) level grater than 15.45%. RDW levels higher than 15.5%, electromyography (EMG) severity, and a clinical score higher than three were found to be independently associated with surgery***.***

**Conclusion:**

An elevated RDW value was related to the severity of the electromyogram. RDW may, therefore, be a useful independent predictor for the decision to surgical treatment of CuTS. ***Level of Evidence III, Retrospective Study.***

## INTRODUCTION

Peripheral entrapment neuropathy (PET) frequently occurs as the result of chronic irrigation of the nerve or compression of the nerve through its passage within the soft tissue and/or channels.^1^ ^,^ ^2^ Cubital tunnel syndrome (CuTS) encompasses all signs and symptoms caused by the chronic impingement of the ulnar nerve at the level of the elbow. The chronic impingement develops following chronic inflammation on ulnar nerve.^3^ ^-^ ^8^ There are four regions of the elbow where the ulnar nerve can suffer compression: first, the retroepicondylar groove (which accounts for the vast majority of cases), second, the humeroulnar arcade (which accounts for 25% of cases), third, the medial intermuscular septum, and finally, the point of exit from the flexor carpi ulnaris.^9^ ^,^ ^10^ CuTS ranks second among PETs after carpal tunnel syndrome (CTS).^3^ ^-^ ^6^ It frequently presents an idiopathic etiology. However, among the secondary etiologies, cubitus valgus, osteoarthritis, subluxation of the ulnar nerve, stenosis caused by fractures, and facial adhesions around the elbow joint have all been pointed to as possible causes.^2^ ^,^ ^4^ The incidence of CuTS has been estimated at 25 cases per 100,000 person-years, and the disease is almost twice as common in males as in females.^4^ The parameters used in the decision-making process for either medical or surgical treatment of patients diagnosed with CuTS include the clinical history, a physical examination, electrodiagnostic studies, and radiological imaging.^2^ ^,^ ^5^ However, the specific hematological parameters used in the decision-making process have not been reported to date.^2^ ^-^ ^4^ ^,^ ^11^ Previous studies have clearly shown that some hematological parameters have been used as predictive markers for a decision to operate in other diseases.^12^ ^-^ ^14^

The red cell distribution width (RDW), reported on all standard haemograms, is an automated measure of the variation in red blood cell (RBC) size or volume. Values are expressed as percentages, with a normal reference range of 11.5 to 14.5%. An elevated RDW indicates a greater variability in the size of the circulating RBCs (anisocytosis) and can occur in patients with nutritional deficiencies, haemoglobinopathies, and haemolysis.^15^ In addition, RDW is associated with cardiovascular disease,^15^ ^-^ ^20^ acute cholecystitis,^14^ acute stroke,^21^ celiac disease,^22^ ankylosing spondylitis (AS),^23^ and mortality in patients with hip fractures treated with a partial prosthesis.^24^ RDW as inflammation marker is associated with many diseases and chronic inflammation condition. The literature, however, does not show any relationship between orthopaedic conditions, PET, and RDW.

In the present study, EMG and clinical assessment have been considered the best surgical decision making parameters for CuTS patients. We hypothesized that RDW as inflammation marker may be used as a predictive marker for the decision to operate in CuTS patients related to the severity of the electromyogram. For that reason, we aimed to investigate whether haemogram parameters are indeed predictive factors for the severity of the disease and also make an alternative decision of surgical treatment in patients with an established diagnosis of CuTS.

## MATERIALS AND METHODS 

The medical files of patients with a diagnosis of CuTS who were either followed-up conservatively or who underwent surgery between July 2008 and April 2015 were screened retrospectively. The study was approved by the Local Hospital Management Committee. Additionally all patients approved the consent form. The diagnosis was based on the patient's history and a clinical examination; i.e. a positive Tinel's sign, sensory loss in the area innervated by the ulnar nerve, pain over the medial epicondyle, weakness of the muscles innervated by the ulnar nerve, and a positive elbow flexion test. Secondary conditions, which would lead to ulnar nerve involvement, were not detected in any participant. The presence of bone pathology (if any) was evaluated using anteroposterior and lateral elbow radiograms. 

Conservative methods (i.e. modification of activity, use of non-steroidal anti-inflammatory drugs and night splints) were tried preoperatively in all patients for at least 6 months. However, some patients were considered for surgery because of the presence of clinically progressive symptoms, sensorimotor deficits, lack of clinical and electroneurographic improvements and a worsening of the objective findings at follow-up controls performed several weeks after the initial visit. Besides, the patients were considered lack of motor function for surgical decision making process.

Patients were excluded from the study if they had inflammatory disorders, an acute infection, had taken local or systemic steroids, had been morbidly obese, were diagnosed with any secondary CuTS pathology, had been diagnosed with cancer, were currently receiving radiotherapy or chemotherapy, had been diagnosed with a psychiatric disorder, had a neurological disease, or had been diagnosed with any cervical pathology.

Patients were asked to complete the 'disability of arm shoulder and hand' (DASH) score. The electromyogram (EMG) measurements of all patients, which included sensory and motor nerve conduction velocity measurements, were evaluated retrospectively.^1^

Haemogram parameters, such as white blood cell count (WBC), haemoglobin (Hb), haematocrit (Htc), mean platelet volume (MPV), neutrophils (NEU), lymphocytes (LYM), platelets (PLT), NEU/LYM, PLT/LYM, and RDW values, which were measured simultaneously with the EMG examination, were recorded. Based on the results of previous studies, the patients' RDW values were divided into subgroups according to the RDW quartiles.^18^

### Statistical Analysis

The data analysis was performed using the Statistical Package for the Social Sciences software, version 15 for Windows (SPSS Inc., Chicago, IL). The data are shown as means ± standard deviation for continuous variables, medians (minimum-maximum) for ordinal variables, and frequencies with a percentage for categorical variables. Comparisons between groups were performed using a one-way analysis of variance (ANOVA), with post hoc analysis by Tukey's honest significant difference (HSD) test, and a t-test plus the Kruskal-Wallis tests or a Mann-Whitney U test for the independent samples for normally and abnormally distributed data, respectively. The categorical variables were analyzed using a chi-squared test. Correlation was evaluated by the Spearman's correlation test. Receiver operator characteristic (ROC) curve analysis was performed to identify the optimal cut-off point of RDW (when the sensitivity and specificity would be maximal) at which to predict the decision for surgery in CuTS patients. Areas under the curve (AUC) values were calculated as measures of the accuracy of the tests. We compared the AUCs using the Z test. We used univariate logistic regression analysis to quantify the association of variables with the decision to operate in CuTS. Variables that were found to be statistically significant in the univariate analysis and other potential confounders were used in a multiple logistic regression model with the forward stepwise method to identify independent prognostic factors of the decision to operate in CuTS. A *p*-value <0.05 was considered to indicate statistical significance.

## RESULTS

**Figure 1:**
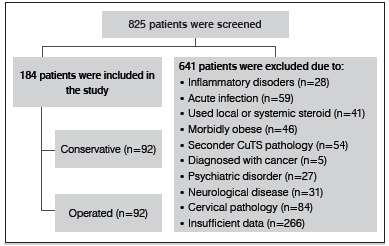
The algorithm and planning criteria of the study.

In the present study, the medical files of 825 patients who underwent EMG examinations between July 2008 and April 2015 were evaluated retrospectively. The patients either underwent surgery (n=124) or were followed-up conservatively. The algorithm and planning criteria of the study are shown in Figure 1. 

The medical files of the patients who met our study criteria and were either followed-up conservatively (n=92) or operated on (n=92) were examined retrospectively. In those patients who were examined, a positive Tinel's sign, sensory loss in the area innervated by the ulnar nerve, pain over the medial epicondyle, weakness of the muscles innervated by the ulnar nerve, and a positive elbow flexion test were found to be more prevalent in the surgery group. In addition, a statistically significant difference was detected between the DASH scores of the groups (*p*<0.001). In the conservative group, low (n=77; 83.7%), and moderate (n=15; 16.3%) EMG intensities were detected, while in the operative group, moderate (n=69; 76.1%), and severe (n=22; 23.9%) intensities were found, and a statistically significant intergroup difference was determined (*p*=0.00). In all patients a statistically significantly positive correlation was detected between the DASH score and the EMG intensity (r=0.911, *p*<0.001). (Table 1)

**Table 1:** The patient demographics and clinical data of both groups.

	Conservative	Operated	*p* Value
Age	46.14 (±15.1) (19-80)	48.4 (±12.68) (21-83)	0.287
Female/Male (%)	%38 / 62%	41.3% / 58.7%	0.652
L / R (%)	65.2% / 34.8%	59.8% / 40.2%	0.448
DASH score	23.82 (±6.76)	59.82 (±5.63)	<0.001
Mild/Moderate/ Severe EMG	83.7% / 16.3% / 0%	0% / 76.1% / 23.9%	0.00

L: left; R: right; DASH: disability of arm shoulder and hand; EMG: electromyogram.

Amongst the haemogram parameters examined, only the RDW values were found to be higher in the surgery group. RDW, whose value as an indicator of high risk has been demonstrated in previous studies, was, based on two cut-off values, evaluated to be >15.5 and ≤15.5. In the conservative group its predictive values were 65.2% (n=60) and 34.8% (n=32) when cut-off values of ≤15.5 and >15.5 were taken into consideration. However, in the surgery group the predictive values of RDW were found to be 48.9 % (n=45) and 51.1% (n=47) at cut-off values of ≤15.5 and 15.5, respectively, with a statistically significant intergroup difference (*p*=0.026). (Table 2) 

**Table 2:** The patient haemogram results of both groups.

	Conservative	Operated	*p* Value
WBC	7.05 (±1.9)	7.43 (±1.71)	0.151
Hb	14.21 (±1.55)	13.97 (±1.7)	0.323
Hct	42.21 (±4.49)	41.63 (±4.6)	0.387
MPV	7.73 (±0.94)	7.93 (±1.17)	0.194
NEU	4.13 (±1.63)	4.36 (±1.38)	0.291
LYM	2.29 (±0.84)	2.42 (±0.77)	0.294
PLT	261.21 (±59.89)	275.22 (±66.95)	0.136
NEU/LYM	2 (±1.3)	2 (±1.35)	0.99
PLT/LYM	125.21 (±55.45)	126.22 (±52.51)	0.900
RDW	15.54 (±1.55)	14.8 (±2.06)	0.008
RDW-Quartile	1.34 (±0.47)	1.51 (±0.5)	0.025

WBC: White blood cell count; Hb: Hemoglobin; Htc: Hematocrit; MPV: Mean platelet volume; NEU: Neutrophil; LYM: Lymphocyte; PLT: Platelet; RDW: Red cell distribution width.

**Figure 2:**
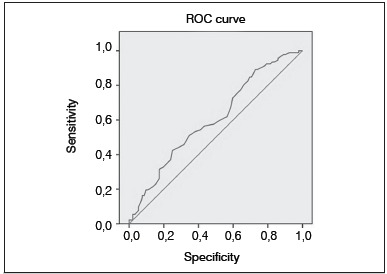
ROC curve analysis of RDW.

In the correlation analysis of patients who were conservatively and surgically treated, we detected a positive correlation between the intensity of EMG, RDW, and RDW-Q in the surgery group alone (RDW; r=0.633, *p*=0.00) (RDW-Q; r=0.784, *p*=0.00). Again, and only in the surgery group, a statistically significantly positive correlation was detected between the DASH score and RDW (r=0.160, *p*=0.030). ROC curve analysis revealed a 0.665 [95% confidence interval (CI) 0.523-0.686, *p*=0.014] area under the curve, 76.3% sensitivity and 84.8% specificity at the cut-off of an RDW level >15.45%. (Figure 2)

In both the univariate logistic regression analyses and the multivariate regression analysis, RDW levels >15.5%, EMG severity, and a clinical score >3 were determined to be independently associated with a decision for surgery (odds ratio [OR] 1.751; 95% CI 1.298-2.684; *p*= 0.008; OR 1.965; 95% CI 1.205-3.098; *p*= 0.001, and OR 1.586; 95% CI 1.195-2.098; *p*=0.003; respectively).

## DISCUSSION

Some physical examination findings-including a positive Tinel's sign, and some tests to measure sensory loss in the area innervated by the ulnar nerve, pain over the medial epicondyle, weakness of the muscles innervated by the ulnar nerve, and a positive elbow flexion test-were reported to be more severe in patients with a presumptive diagnosis of CuTS who were scheduled for surgery.^2^ ^,^ ^4^ ^,^ ^5^ In the present study, these findings were determined to be more prominent in patients in the surgery group. In patients with a diagnosis of CuTS, the DASH scoring system is an important guiding tool in the determination of the daily functions of the patient. In addition, it has been recognized that DASH score is higher in CuTS patients requiring surgery, whilst it drops during the postoperative period.^25^ ^-^ ^27^ We also detected statistically significant higher DASH scores in patients who underwent surgery when compared with the conservatively treated group. As an objective diagnostic criterion, EMG is important for making a definitive diagnosis in patients with presumptive PET. Indeed, EMG aids in the determination of the level, severity, and type of nervous involvement.^1^ ^,^ ^6^ ^,9^ In the decision-making process for treatment alternatives in CuTS patients, the presence of a correlation has been reported between the clinical manifestations of the patient and the intensity of the EMG.^1^ ^,^ ^3^ ^-^ ^5^ ^,^ ^9^ ^,^ ^11^ In patients diagnosed with CuTS, in cases where transmission of stimuli slows as demonstrated on the EMG, the treatment of CuTS has reportedly changed from a conservative approach to ulnar neurolysis, decompression, anterior transposition, and epicondylectomy.^1^ ^,^ ^5^ ^,^ ^11^ ^,^ ^25^ ^,^ ^26^ In the present study, we also applied the decompression procedure in cases with a moderate intensity of EMG, and anterior transposition for those with a severe EMG intensity. In all cases for surgery a statistically significant positive correlation existed between the DASH score and the EMG intensity.

A specific haemogram predictor marker for the diagnosis of CuTS has not yet been reported. However, some haemogram parameters are known to have predictive values for various diseases, including some involving the musculoskeletal system.^15^ ^,^ ^17^ ^,^ ^21^ ^-^ ^24^ ^,^ ^28^ ^-^ ^33^ Balbaloglu et al.^29^ reported increased MPV blood levels as a predictive marker for osteoarthritis. However, Li et al*.* ^30^ demonstrated that MPV was negatively associated with bone mineral density in postmenopausal females. Kisacik et al*.* ^31^ indicated the blood MPV level to be an inflammatory parameter in patients with AS or rheumatoid arthritis (RA). Tutoglu et al*.* ^32^ detected a correlation between the diagnosis of CTS and the MPV in the geriatric age group. Fu et al.^33^ indicated that the NEU/LYM and PLT/LYM ratios were important predictors in the evaluation of disease activity in RA. Boyraz et al.^34^ indicated that the NEU/LYM and PLT/LYM ratios were important evaluation of disease activity in RA treatment with anti-TNF agents. Besides, RDW show important predictors in the evaluation of other disease that is aortic valve implantation as a poor prognostic marker,^17^ use as an indicator in coeliac disease.^22^ RDW may be useful in predicting the severity and functional outcomes of a stroke in acute stroke patients who have had symptoms for <24 hours,^21^ and may also be a predictive tool in many other diseases, as is seen in RA patients who have one of the modifiable cardiovascular risk factors.^15^ In a study by Peng et al*.* ^23^ RDW was reported as a marker of disease activity in AS. Zehir et al*.* ^24^ noted the role of RDW in predicting mortality in patients with hip fractures. Chronic compression may be due to prolonged pressure of the medial edge of the elbow on the ulnar nerve bed and the initial inflammatory reaction of the idiopathic CuTS.^7^ ^,^ ^8^ In inflammatory conditions that develop following chronic compression, RDW is one of the haemogram parameters that can indicate the inflammatory process. We considered the above mentioned studies about RA, AS and other inflammatory disease as guidance for the present study and investigated whether any haemogram marker is a predictor in patients diagnosed with CuTS. We also detected statistically significantly higher RDW values in CuTS patients who received surgical treatment.

There were some limitations to our study. The number of patients enrolled in the study was small; therefore, our findings should not be generalized. Instead, they should be supported by further studies using a sufficient number of patients. The retrospective nature and one-centre design of this study were other limitations. In addition, vitamin B12 levels of blood were not screened with haemogram parameters. Because differential central and peripheral neuropathy syndromes was seen in the vitamin B12 deficit, this correlated with vitamin B12 deficit and axonal processes were not reflected by the present study, and design of this study were other limitations. Prospective future studies conducted using a multicentre approach, vitamin B12 levels and haemogram parameters are needed for more accurate results.

## CONCLUSION

In conclusion, EMG and clinical assessment have been considered the best surgical decision making parameters for CuTS patients. However, RDW which is one of the haemogram parameters that can indicate the inflammatory process is an important parameter in inflammatory conditions that develop following chronic nerve compression. Therefore, an elevated RDW value is related to the severity of the chronic nerve compression. RDW may be a useful independent predictor for a decision to operate in patients with CuTS.
